# A Novel Aptamer Biosensor Based on a Localized Surface Plasmon Resonance Sensing Chip for High-Sensitivity and Rapid Enrofloxacin Detection

**DOI:** 10.3390/bios13121027

**Published:** 2023-12-13

**Authors:** Pan Wang, Liyun Ding, Yumei Zhang, Xingdong Jiang

**Affiliations:** 1 National Engineering Research Center of Fiber Optic Sensing Technology and Networks, Wuhan University of Technology, Wuhan 430070, China; 317552@whut.edu.cn (P.W.); 307462@whut.edu.cn (Y.Z.); 2 School of Physical Science and Technology, Lanzhou University, Lanzhou 730000, China; jiangxd@lzu.edu.cn

**Keywords:** enrofloxacin, AuNPs-Apt complex, local surface plasmon resonance, sensing chip

## Abstract

Enrofloxacin, a fluoroquinolone widely used in animal husbandry, presents environmental and human health hazards due to its stability and incomplete hydrolysis leading to residue accumulation. To address this concern, a highly sensitive aptamer biosensor utilizing a localized surface plasmon resonance (LSPR) sensing chip and microfluidic technology was developed for rapid enrofloxacin residue detection. AuNPs were prepared by the seed method and the AuNPs-Apt complexes were immobilized on the chip by the sulfhydryl groups modified on the end of the aptamer. The properties and morphologies of the sensing chip and AuNPs-Apt complexes were characterized by Fourier transform infrared spectroscopy (FTIR), UV-Vis spectrophotometer, and scanning electron microscope (SEM), respectively. The sensing chip was able to detect enrofloxacin in the range of 0.01–100 ng/mL with good linearity, and the relationship between the response of the sensing chip and the concentration was Δλ (nm) = 1.288log ConENR (ng/mL) + 5.245 (R^2^ = 0.99), with the limit of detection being 0.001 ng/mL. The anti-interference, repeatability, and selectivity of this sensing chip were studied in detail. Compared with other sensors, this novel aptamer biosensor based on AuNPs-Apt complexes is expected to achieve simple, stable, and economical application in the field of enrofloxacin detection.

## 1. Introduction

The discovery of penicillin from mold in the 1920s marked a pivotal moment in human history, ushering in a transformative era in antimicrobial medicine [1,2]. Since then, fluoroquinolone antibiotics like enrofloxacin have become widely utilized for treating bacterial and mycoplasma infections in animals [3,4]. However, the abuse of these antibiotics can have far-reaching implications for human health and the ecosystem, potentially leading to intestinal dysbiosis, fetal malformations, and the development of antibiotic resistance, which poses significant challenges to disease treatment [5,6,7]. The World Health Organization (WHO) has announced the detection of fluoroquinolone antibiotic residues in humans and reported increased resistance to antibiotics in human bacterial infections, according to an analysis by the Global Monitoring System for Antimicrobial Resistance and Use (GLASS) [8,9]. Therefore, stringent regulations on antibiotic usage and maximum residue limits have been implemented by numerous countries and organizations to safeguard public safety and ecosystem integrity [10,11].

Traditional methods for the detection of enrofloxacin include fluorescence quenching [12,13,14], electrochemical methods [15,16], and immunological methods [17]. Despite their widespread application, these methods are encumbered by costly detection equipment, complicated pretreatment operations, and the requirement for specialized personnel, compared with optical sensors [18,19], which greatly limit the efficiency of detection. Consequently, there is a compelling need to develop a highly sensitive, rapid, convenient, and reliable method for detecting enrofloxacin residues. Plasmon sensors have already been developed for a wide range of applications in the field of sensing due to their extremely high sensitivity. Muhammad A. Butt et al. proposed a design of a raceway-integrated circular cavity based on a metal–insulator–metal waveguide and optimized with the finite element method for refractive index sensing [20]. Yuan-Fong Chou Chau et al. proposed a plasmonic perfect absorber based on an array of metal nanorods for ultrasensitive refractive index sensing in the near-infrared region [21]. Vasyl G. Kravets et al. investigated a metal–dielectric–graphene hybrid heterostructure and found that the properties of these heterostructures can have a significant impact on the sensitivity of surface plasmon resonance biosensing [22]. Kandammathe Valiyaveedu Sreekanth et al. developed a highly sensitive biosensing platform based on hyperbolic metamaterials, which can be excited by a grating coupling technique. The excitation by grating coupling technique is able to obtain extreme sensitivity patterns [23].

Gold (Au) is one of the most chemically stable elements in nature, while nano-gold exhibits distinctive photoelectric, physical, and chemical properties, along with good biocompatibility. Among the numerous investigations on nanomaterials, gold nanoparticles (AuNPs) are one of the most widely and deeply studied nanomaterials by researchers [24]. The particle size distribution of AuNPs ranges from 1 to 100 nm, and they exhibit excellent dispersion in solution. Upon excitation by incident light, the free electrons on the surface of AuNPs induce localized surface plasmon resonance (LSPR), leading to the emergence of characteristic resonance peaks in the UV-visible region [25,26]. The resonance peaks are influenced by the distance between the AuNPs and the changes in the refractive index of the surrounding medium. Furthermore, the substantial specific surface area of AuNPs makes it easy to be functionalized by various ligands such as peptides [27], nucleic acids [28], enzymes [29], and antibodies [30], so it has the ability to specifically recognize analytes.

Among the substances with the ability to functionalize AuNPs, aptamers are extensively used because of their unique physicochemical properties [31]. Aptamers are single-stranded oligonucleotides that can specifically bind to a target molecule generated by screening through SELEX technology (systematic evolution of ligands by exponential enrichment), which has the advantages of in vitro synthesis, a simple synthesis method, low cost, and batch synthesis that does not result in variations [32]. Single-stranded oligonucleotides adopt a specific three-dimensional structure through self-adaptive folding, facilitated by various interaction forces, including nucleotide-base complementary pairing and hydrogen bonding [33]. This three-dimensional structure exhibits specific binding to the target molecule through intermolecular forces. Its binding dissociation constant can reach nanomolar and picomolar levels, comparable to those of monoclonal antibodies. Enhancements and optimizations through end immobilization, sequence optimization, and chemical modification can further improve its applicability. In comparison to antibodies, aptamers exhibit superior affinity, less exacting storage and application requirements, enhanced anti-interference capabilities, and superior synthesis purity, rendering them suitable for application in the field of biosensing [34]. AuNP-based aptasensors have now become a research hotspot and thus have been widely studied and applied [35,36], especially in antibiotic detection [37]. Li Gao et al. developed a new colorimetric sensor based on aptamer and gold nanoparticles (AuNPs) to detect cocaine, and optimized the sensing system by using MoS_2_ [38]. Shaoming Yang et al. used aptamer modified on a glassy carbon electrode treated with reduced graphene oxide (rGO) and AuNPs to detect thrombin [39]. Ying Jiang et al. developed a colorimetric sensor for detecting exosomes of cancer markers, and the aptamer played a dual role in protecting AuNPs and specifically recognizing molecular substances [40].

In this paper, a novel aptamer biosensor based on a LSPR sensing chip with a AuNPs-Apt complex was developed which was used specifically for enrofloxacin detection. The characteristic resonance peaks could be shifted with the change of refractive index near the AuNPs when enrofloxacin specifically binds to the aptamer on the chip surface. At the same time, a set of LSPR sensing systems was assembled by using a light source, spectrometer, microfluidic device, and optical coupling platform to enhance the practical application ability of the sensing chip. The effects of various factors on the performance of the sensor were individually analyzed to optimize the functionality of the sensing chip. The sensing chip demonstrates excellent linearity within the concentration range of 0.01–100 ng/mL, exhibiting a high level of sensitivity.

## 2. Experimental Section

### 2.1. Materials and Reagents

Gold chloride trihydrate (HAuCl_4_), tris (2-carboxyethyl) phosphine (TCEP), trisodium citrate, (3-aminopropyl) trimethoxy silicon (APTMS), sodium chloride (NaCl), and potassium chloride (KCl) were purchased from Aladdin Chemical Co., Ltd. (Shanghai, China). HCl (38%), and HNO_3_ (68%), H_2_SO_4_, hydrogen peroxide (H_2_O_2_), and anhydrous ethanol were purchased from Sinopharm Chemical Reagents Beijing Co., Ltd. (Shanghai, China). Enrofloxacin (ENR), ofloxacin (OFLX), pefloxacin (PEF), norfloxacin (NFX), and ciprofloxacin (CIP) were purchased from Macklin Biochemical Co., Ltd. (Shanghai, China). Ultra-pure water used in the experiment was provided by the Hitech-K flow water purification system.

The ENR aptamer (5′-SH-CCCATCAGGGGGCTAGGCTAACACGGTTCGGCTCTCTGAGCCCGGGTTATTTCAGGGGGA-3′) [41] was synthesized by Shanghai Sangon Biotechnology Co., Ltd. (Shanghai, China).

### 2.2. Preparation of AuNPs

AuNPs with different particle sizes were prepared by the seed method [42]. Briefly, HAuCl_4_ was employed as the gold source, while trisodium citrate served as the reducing agent. The seed particles were obtained by heating a mixed solution comprising 200 μL of HAuCl_4_ solution (20 mM) and 30 mL of trisodium citrate solution (2.2 mM) to boiling under oil bath conditions. Subsequently, the seed particles were enlarged through the sequential addition of 200 μL of HAuCl_4_ solution (20 mM) and 400 μL of trisodium citrate solution (60 mM) several times, and, finally, different particle sizes of AuNPs were obtained. The synthesized AuNPs exhibited particle sizes ranging from 20 to 50 nm, and during the reaction, the solution started with a light pink color, then deepened, and, finally, took on a burgundy color. As the particle size increases, the solution becomes darker and cloudy.

### 2.3. Preparation of the LSPR Aptamer Sensing Chip

**Figure 1:**
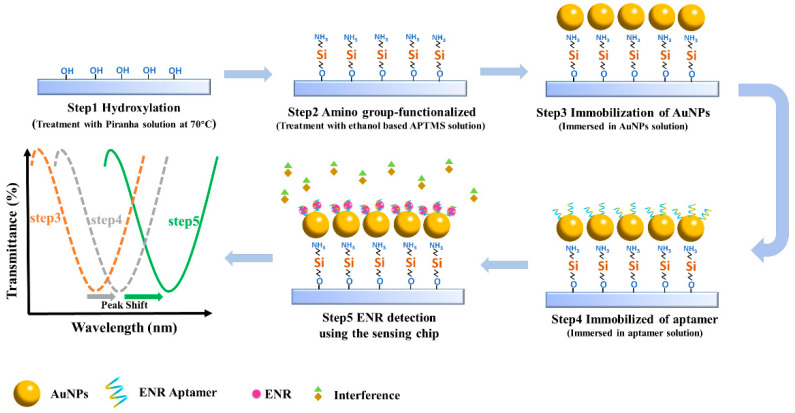
Schematic of the construction of the sensing chip and spectral changes corresponding to each step.

The quartz glass slides were first ultrasonicated with ethanol and then washed with ultrapure water. The surface of the glass slides was hydroxylated by placing it in piranha solution (H_2_SO_4_:H_2_O_2_ = 7:3) for 3 h and then placing it in an oven at 110 °C for 1 h. Following this, the slides were immersed in 5 mL of ethanol-based APTMS (4%) for 20 min, and washed and baked in an oven at 60 °C for 3 h to functionalize the surface with amino groups. Then, the amino group-functionalized slips were incubated with 100 μL AuNPs solution so that the AuNPs were fixed on the surface of the glass slides. Finally, the glass chips were functionalized with 100 μL aptamer solution and washed with ultrapure water to remove unbound aptamer. The aptamer binds to the AuNPs on the chip by forming the Au-S bond. The process is shown in Figure 1. It was also observed that the characteristic peaks of the corresponding transmission spectra drifted at different steps, the exact principle of which will be discussed subsequently in the ENR detection section. The microfluidic channel was fabricated by mixing dimethyl silicone polymer (PDMS) and curing agent into the mold, followed by heating until the mixture solidified. Specifically, the PDMS and the curing agent were combined at a mass ratio of 10:1 and poured into the mold after being thoroughly stirred. The mold was placed in a vacuum drying oven at 150 °C for 5 h to complete the hardening process, after which the mold was removed and sample entry and exit hoses were inserted. The channel size of the microfluidic device was 1 × 1 × 10 mm.

### 2.4. Construction of the LSPR Sensing Platform

**Figure 2:**
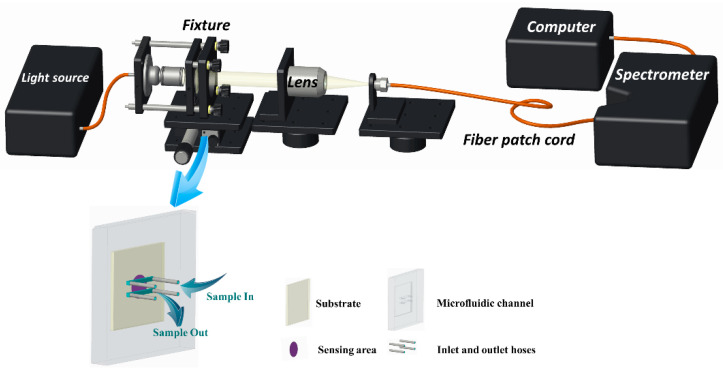
Schematic of the optical sensing platform and the composition of the microfluidic chip.

The optical experimental platform for detecting ENR is shown in Figure 2. The sensing chip is embedded in the microfluidic device and secured in the sensing position of the system using a fixture. After turning on the light source, the position of the focused light is adjusted through the optical coupling platform to ensure its arrival at the sensing area of the chip. Then, the transmitted light is focused through the lens, coupled to the fiber patch cord, and, finally, transmitted to the spectrometer for final data reception and processing.

### 2.5. ENR Detection

Standard solutions of ENR at various concentrations were prepared using PBS buffer solution and sonicated to achieve uniform dispersion. A syringe pump was used to introduce the liquid into the microfluidic channel, ensuring contact with the sensing area on the chip surface. The light source was then turned on to allow light to pass through the sensing area, and the changes in the characteristic peaks in the spectra were observed. The duration from the start of detection to the stabilization of the peak changes constitutes the assay time of the sensor. As shown in Figure 1, the positions of the characteristic peaks shifted after the functionalization treatment, indicating that the aptamer successfully binds to the AuNPs. When the ENR standard solution is introduced, the aptamer can be folded through hydrogen bonding and specific recognition, thus forming the aptamer-ENR complex. This process results in a change in the medium environment near the AuNPs, thereby leading to alterations in the positions of the characteristic peaks. The standard curve is established based on the relationship between the detected ENR concentration and the characteristic peak drift.

### 2.6. Characterization

The distribution of AuNPs-Apt complexes on the chip surface was characterized by a JSM-IT800 (Japan Electronics Co., Kanagawa, Japan) scanning electron microscope (SEM), and the elemental content of the sensing chip surface before and after functionalization was analyzed using the Ultim Max 65 energy dispersive spectrometer (EDS). The UV-visible spectra were collected by a UV-2450 UV-visible spectrophotometer (Shimadzu Co., Tokyo, Japan). The FTIR spectra of the AuNPs and AuNPs-Apt complexes in the range of 600–4000 cm^−1^ were obtained by a Nicolet 6700 FTIR spectrometer (Thermo Fisher Co., Waltham, MA, USA) The sensing system was constructed using a Flame-S-VIS-NIR-ES spectrometer (Ocean Optics Co., Orlando, FL, USA) and light source, as well as various types of optical coupling platforms. The zeta potential of the AuNPs as well as the AuNPs-Apt complexes was obtained by measuring with a Malvern Nano-ZS Zeta Potentiometer (Spectris Co., Shanghai, China).

## 3. Results and Discussion

### 3.1. Characterization of the AuNPs-Apt and LSPR Sensing Chip

**Figure 3:**
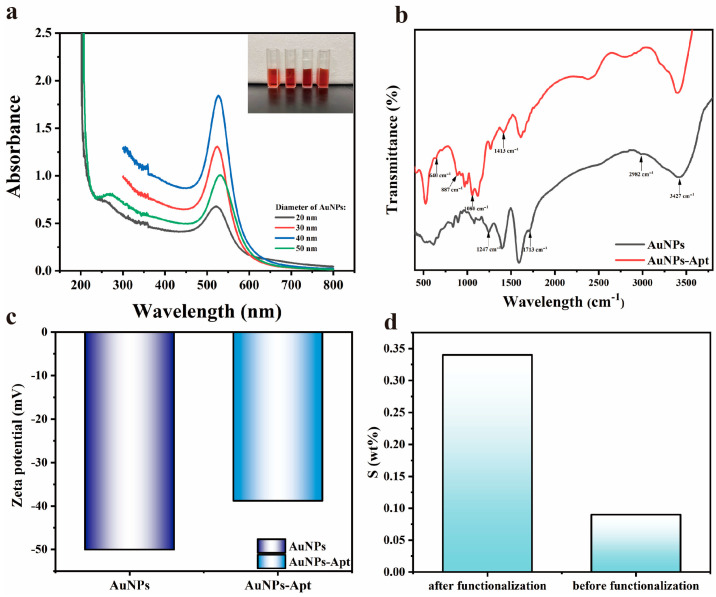
(**a**) UV-Vis absorption spectra of AuNPs with different particle sizes. Inset: the physical picture of AuNPs; (**b**) FT-IR spectra of AuNPs before and after functionalization; (**c**) zeta potential of AuNPs before and after functionalization; (**d**) content of S element in sensing chip before and after functionalization.

The UV-visible absorption spectra were utilized to measure the absorption spectra of AuNPs with different particle sizes, and the results are shown in Figure 3a. By referring to the intensity and position of absorption peaks, the diameter of AuNPs can be calculated to be about 20–50 nm [43]. The Zeta potential shown in Figure 3c illustrates that the AuNPs exhibit electronegativity attributed to the C_6_H_7_O_7_^−^ on its surface, indicating the stability and excellent dispersion of AuNPs. After the functionalization treatment, the electronegativity of AuNPs-Apt decreased, indicating that the surface of AuNPs was bound with aptamers and changed its electronegativity. In addition, we studied the cytotoxicity of AuNPs-Apt, and the experimental steps and the discussion of the results are supplied in the Supplementary S1 part of the supporting information.

The FTIR spectra show the functional groups on the surface of AuNPs, and the results are shown in Figure 3b. For the AuNPs grown by the seed method, there are characteristic peaks generated by -OH stretching vibration (3427 cm^−1^), -CH_2_ antisymmetric stretching vibration (2982 cm^−1^), C=O stretching vibration (1713 cm^−1^), and C-O stretching vibration (1247 cm^−1^) due to the participation of trisodium citrate. After aptamer treatment, the characteristic peaks attributed to the exchange vibration of purines and pyrimidines (1413 cm^−1^), symmetry vibration of PO_2_^−1^ (1080 cm^−1^), cyclic vibration of alkylpentoses (887 cm^−1^), and C-S telescoping vibration (640 cm^−1^) appeared as a result of the binding of aptamer on the surface of the AuNPs. The presence of these characteristic peaks indicated the exceptional stability of prepared AuNPs and the successful binding of the aptamer to the AuNPs [44].

**Figure 4:**
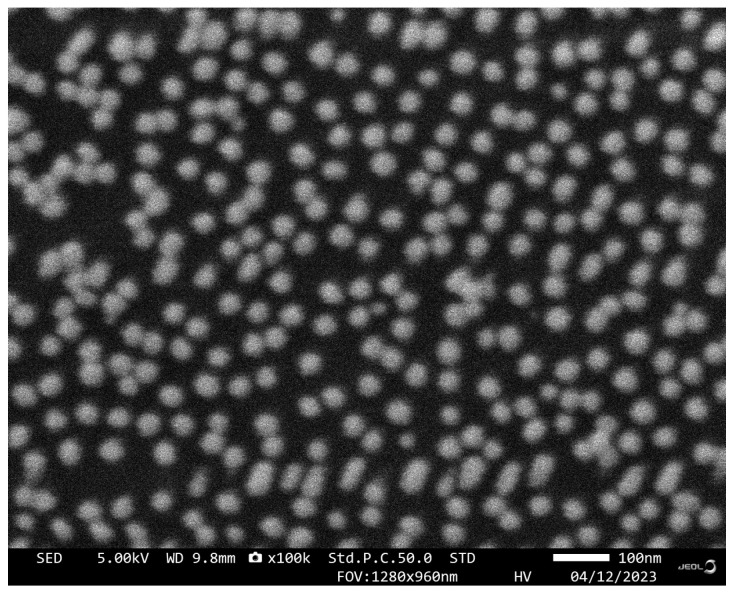
SEM image of the sensing chip.

The sensing chip was characterized by SEM, and the results are shown in Figure 4. After AuNPs incubation and aptamer functionalization, AuNPs-Apt complexes formed on the surface of the glass slide are very uniformly distributed without agglomeration, exhibiting a relatively homogeneous particle size distribution of approximately 40 nm. From the results of the EDS spectra shown in Figure 3d, it can be seen that the sulfur content changed significantly before and after functionalization, indicating that the AuNPs on the chip were successfully modified by the aptamer.

### 3.2. Optimization of the Experimental Conditions

In order to obtain the best ENR detection performance, the size of AuNPs, the incubation time of AuNPs, and the concentration and the processing time of aptamer were investigated. The size and the incubation time of AuNPs were investigated based on the refractive index sensitivity obtained by testing NaCl solutions with different refractive indexes. When exploring the concentration and the processing time of aptamer, we used the sensitivity obtained by testing different concentrations of ENR solution.

**Figure 5:**
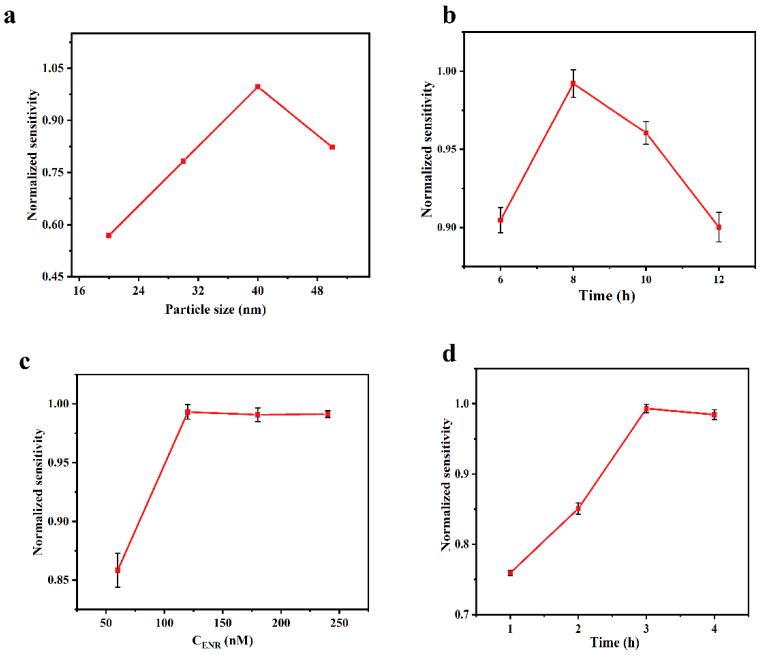
Effect of different experimental conditions. (**a**) Diameter of AuNPs; (**b**) incubation time of AuNPs; (**c**) concentration of ENR aptamer; (**d**) time of functionalization treatment.

According to Mie theory and Maxwell-Garnett theory [45,46], the position and shape of the LSPR characteristic peaks in the UV-Vis region are affected by the size as well as the shape of the AuNPs particles, so we investigated the relationship between the particle size and the sensitivity of the sensing chip in the preparation process, with the results shown in Figure 5a. The refractive index sensitivity of the sensing chip exhibits a trend of increasing and then decreasing with the increase in AuNPs particle size, reaching its peak at a particle size of 40 nm. The relationship between the incubation time of AuNPs and the refractive index sensitivity of the sensing chip was investigated under the optimal particle size condition (40 nm), and the results are shown in Figure 5b. The sensitivity increases and then decreases with the increase in incubation time. This is because, when the incubation time is short, the number of AuNPs immobilized on the chip is insufficient, producing a weak LSPR effect. As the incubation time increases, the number of AuNPs fixed on the chip also increases, leading to an enhancement of the LSPR effect. However, when the number of AuNPs increased to an excessive amount, it led to agglomeration, which significantly reduced the sensitivity of the sensing chip. The optimum incubation time was 8 h.

The concentration and the processing time of aptamer in the functionalization step will directly affect the degree of aptamer coverage on the chip surface, thus impacting the detection ability of the sensing chip. The sensing chip was functionalized with aptamer at concentrations ranging from 60 to 240 nM. As shown in Figure 5c, the sensitivity of the sensing chip for the detection of ENR increased with the rise of aptamer concentration and stabilized after reaching 120 nM. This suggests that the aptamer binding was saturated at this point, and thus the optimal concentration was 120 nM. Having investigated the concentration of aptamer, we then explored the optimal processing time under this condition, and it can be observed from Figure 5d that the detection sensitivity of ENR increased with the increase in the processing time and stabilized after 3 h.

### 3.3. ENR Analysis Using Standard Solutions

**Figure 6:**
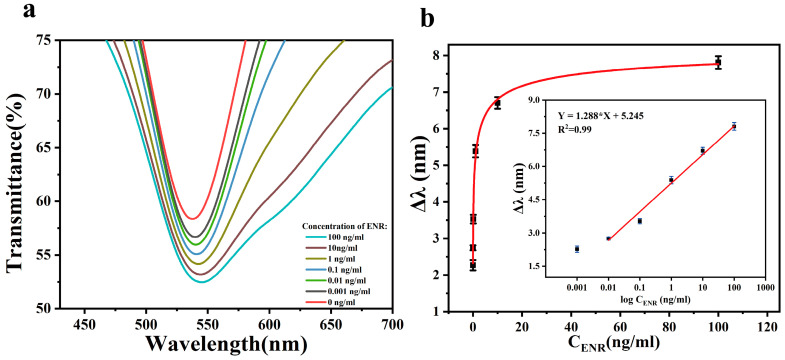
(**a**) The transmission spectra of the sensing chip in the presence of ENR solution with different concentrations ranging from 0.001 to 100 ng/mL; (**b**) effect of different concentrations of ENR on Δ*λ*. Inset: the calibration curves.

After optimizing the various conditions for the preparation of the sensing chip, we further investigated its ability to detect ENR. The standard solution of ENR with a concentration ranging from 0.001–100 ng/mL was injected into the sensing area of the chip through the microfluidic channel using an injection pump. The change in the characteristic peak position in the spectra was then detected, with the result shown in Figure 6a. When the ENR binds to the aptamer on the sensing chip, the refractive index near the AuNPs changes, resulting in the displacement of the peak position of the characteristic peak in the spectra (Δ*λ*). As shown in Figure 6b, there is no linear relationship between Δ*λ* and ENR concentration, so the concentration was logarithmically processed and then linearly fitted. At the ENR concentration of 0.001 ng/mL, the deviation of Δ*λ* from the fitted curve is large, so the limit detection (LOD) of the sensor is 0.001 ng/mL. By analyzing the relationship between Δ*λ* and the ENR solution in the concentration range of 0.01–100 ng/mL, the linear regression equations were obtained as follows: Δ*λ* (nm) = 1.288log Con_ENR_ (ng/mL) + 5.245 (R^2^ = 0.99). The minimum detectable increment of the sensor is 0.001 ng/mL. This indicates that the sensing chip exhibits a high sensitivity and a wide detection range for ENR.

In addition, the comparison results of the sensing chip with some previously reported ENR detection methods are presented in Table 1. The results show that the detection method we provided can combine both high and rapid sensitivity, as well as a wide detection range, and a good linear relationship. For example, compared to electrochemical sensors, it is less susceptible to other factors, the operation process is simpler, and the assay time is shorter.

### 3.4. Interference Immunity of the LSPR Sensing Chip

We evaluated the practical application capability of the sensing chip by assessing its resistance to interference. The interferents and the ENR solution to be tested were prepared into a mixed solution with a mass ratio of 10:1 and then detected by the sensing chip. The anti-interference ability of the sensing chip was defined by detecting the interference rate under different interferents, with the results shown in Table 2. The interference ratio is defined as

$$I.R.=\frac{\left(C-C_{0}\right)}{C_{0}}*100\%$$

where *C*_0_ is the concentration of the configured enrofloxacin solution and *C* is the actual enrofloxacin concentration detected by the sensor. The interference rate is less than 3%, which suggests a strong anti-interference capability. This is attributed to the occupation of the binding sites on the surface of AuNPs following the formation of aptamer ENR, enabling the sensor to exhibit good anti-interference ability without being affected by other substances.

### 3.5. Comprehensive Performance of the LSPR Sensing Chip

**Figure 7:**
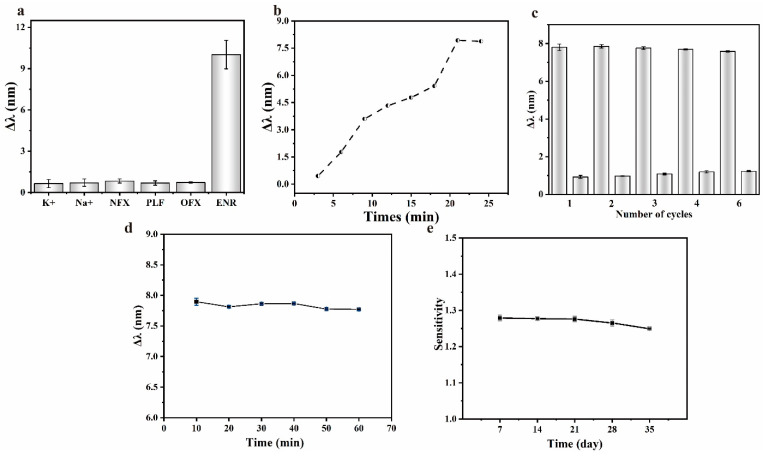
(**a**) Selectivity of different interferents of the sensing chip; (**b**) response time of the sensing chip; (**c**) repetitive detection ability of the sensing chip; (**d**) stability of the response of the sensing chip during detection; (**e**) stability of the sensing chip during storage.

The comprehensive performance of the sensing chip was evaluated by exploring the response time, repeatability, stability, and selectivity. Standard solutions of several fluoroquinolone antibiotics similar in structure to ENR were prepared at the same concentration to determine the selective detection ability of the sensing chip, with the results shown in Figure 7a. The sensing chip responded weakly to substances other than ENR, indicating that ENR could be specifically detected. To evaluate the response time, spectra were recorded at three-minute intervals upon the injection of the test liquid into the sensing region of the chip, with the results shown in Figure 7b. The response of the sensing chip gradually increased and eventually stabilized after 21 min, indicating a response time of approximately 20 min.

The repeatability of the sensing chip was evaluated by repeated measurements of the ENR solution using the same sensing chip. Briefly, after the response had stabilized, the sensing area of the chip was rinsed with the buffer until the characteristic peaks returned to the initial state, and the response of the sensing chip was observed by reintroducing the ENR solution. The above procedures were repeated, and the responses of the sensing chip were recorded, with the results shown in Figure 7c. The stability of the sensing chip during detection and storage was tested. After the response of the sensing chip was stabilized (about 21 min), the timing was restarted and the spectra were recorded over time to assess its stability during detection, as shown in Figure 7d. Unfunctionalized sensing chips were sealed in a nitrogen atmosphere and stored at 4 °C. One chip was taken out every 7 days, functionalized with aptamer, and used to detect the standard solutions of ENR. The stability of the chip after long-term storage was evaluated by observing the change of sensitivity, with the results shown in Figure 7e.

### 3.6. Analysis of ENR in Real Samples

Using tap water to prepare different concentrations of ENR solutions as samples, we evaluated the applicability of the developed aptamer sensing chip to detect ENR in real samples. As shown in Table 3, the recovery was obtained by dividing the concentration calculated from the response of the sensor by the actual concentration added in the solution, which was calculated to be in the range of 91.94–108.38%. Relative standard deviation (RSD) is the ratio of the standard deviation of a set of recovery data to the mean value, reflecting a high degree of accuracy of the test. The result demonstrates that the sensing chip has significant potential application as a sensor in actual sample detection of ENR.

## 4. Conclusions

A set of equipment for ENR detection was prepared with a microfluidic sampling device and an optical coupling platform, where an ENR sensing chip based on a AuNPs-Apt complex was developed. AuNPs with high stability were prepared by the seed method and then bonded to the surface of glass by a chemical cross-linking method. Then, the AuNPs-Apt complex was formed on the chip through an -SH group modified at the end of the aptamer. The complex has good biocompatibility. In the presence of ENR, due to specific recognition and hydrogen bonding, the aptamer can be folded to form an AuNPs-ENR complex, which allows the sensing chip to respond. The standard curve of the sensing chip is Δ*λ* (nm) = 1.288log Con_ENR_ (ng/mL) + 5.245 (R^2^ = 0.99). In conclusion, this aptamer-based LSPR sensor is inexpensive to prepare, simple to operate, and able to simultaneously offer the advantages of high sensitivity, wide detection range, and short assay time, compared with other ENR sensors. It has great potential for in-field rapid detection of ENR residues in aqueous environments.

## Figures and Tables

**Table 1:** Comparison of other different detection methods.

Detection Method	Probe Used	Liner Range	LOD	Assay Time	Reference
Fluorescent biosensor	N-CDs	1~15 μg/mL	0.009 μg/mL	4 min	[12]
Upconversion nanoparticle	CSUNPs	0.976~62.5 ng/mL	0.47 ng/mL	2 h	[47]
Lateral flow immunoassay	AuNPs-Ab	20~50 ng/mL	20 ng/mL	10 min	[48]
SPR	Au	3~20 ng/mL	3 μg/mL	—	[49]
LSPR	PDA-MIP	25~1000 ng/mL	61.1 ng/mL	20 min	[50]
Electrochemistry	CoNi-MOF-AuNPs	10^−6^~100 ng/mL	3.3 × 10^−3^ pg/mL	100 min	[41]
Proposed method	AuNPs-Apt	0.01~100 ng/mL	0.001 ng/mL	20 min	This work

**Table 2:** Detection of ENR in the presence of different interferers by a sensing chip.

Interferent	Amount of Interferents Added (ng/mL)	Addition Amount of ENR C_0_ (ng/mL)	Detection Amount of ENR C (ng/mL)	Interference Ratio (%)
Ciprofloxacin	100	10	10.23	2.3
Pefloxacin	100	10	9.76	−2.4
Norfloxacin	100	10	9.72	−2.8
Ofloxacin	100	10	10.26	2.6
Na^+^	100	10	10.15	1.5
K^+^	100	10	10.19	1.9

**Table 3:** Water samples were added with different concentrations of ENR for recovery tests.

Sample	Added Concentration (ng/mL)	Detected Concentration (ng/mL)	Recovery (%) (n = 3)	RSD (%)
1	0.1	0.09194	91.94	3.26
2	1	1.0838	108.38	4.45
3	10	10.4757	104.75	3.53
4	100	98.0527	98.05	3.51

## Data Availability

Data are contained within the article and Supplementary materials.

## Supplementary Materials

The following supporting information can be downloaded at: https://www.mdpi.com/article/10.3390/bios13121027/s1, S1: Cytotoxicity of AuNPs-Apt. Figure S1: Cytotoxicity results of AuNPs-Apt (100%) and AuNPs-Apt (50%) after 16 and 24 h in-cubation. S2: EDS spectra. Figure S2: (a) EDS spectra after functionalization of sensing chips. (b) EDS spectra before functionalization of sensing chip. Table S1: The elemental contents of the sensing chip after functionalization. Table S2: The elemental contents of the sensing chip before functionalization.
