# Supplementary material for: A Novel Aptamer Biosensor Based on a Localized Surface Plasmon Resonance Sensing Chip for High-Sensitivity and Rapid Enrofloxacin Detection
Source: Biosensors (Basel). 2023 Dec 13;13(12):1027. doi: 10.3390/bios13121027 (PMC10741520; doi:10.3390/bios13121027)
Supplement: Supplementary file 1 [file biosensors-13-01027-s001.zip › biosensors-2729385-supplementary.pdf]

Article

A Novel Aptamer Biosensor Based on a Localized Surface Plasmon Resonance Sensing Chip for High-Sensitivity and Rapid Enrofloxacin Detection

Pan Wang [1], Liyun Ding [1,2,*], Yumei Zhang [1] and Xingdong Jiang [2]

[1] National Engineering Research Center of Fiber Optic Sensing Technology and Networks, Wuhan University of Technology, Wuhan 430070, China; 317552@whut.edu.cn (P.W.); 307462@whut.edu.cn (Y.Z.)
[2] School of Physical Science and Technology, Lanzhou University, Lanzhou 730000, China; jiangxd@lzu.edu.cn
* Correspondence: dingly@lzu.edu.cn

S1. Cytotoxicity of AuNPs-Apt

The CCK8 assay was used to evaluate the cytotoxicity of AuNPs-Apt coupling. The procedure was as follows, HGC-27 cells were inoculated in 96-well cell plates at a rate of 8000 per well with 80 μL of cell suspension, and the cell plates were placed in an incubator at 37°C and 5% CO_2 overnight. 20 μL of complex working solution (AuNPs-Apt complex) was added to the cell plates and cultured in the incubator for 16 and 24 hours protected from light. After the incubation was completed, CCK8 was added to the cells and then incubated in the dark for 1 h at 37°C in an incubator. The absorbance at 480 nm was measured and the inhibition rate was calculated according to the following equation,

$$\text{Cell inhibition rate (\%)} = (1 - OD_S/OD_{NC}) \times 100\%$$

where OD_S in the formula represent the absorbance of the sample wells and OD_{NC} represents the absorbance of the blank control wells. The results were showed t in Fig S1. The inhibitory effect of AuNPs-Apt (100%) on the cells was -3.42 % and -7.25 % at 24 h and 16 h treatment time, respectively, and the inhibitory effect of AuNPs-Apt (50%) on the cells was -2.17 % and -6.08 %, respectively. The complexes were basically non-toxic to cells, indicating that the sensing chip has a broad application prospect in the field of biological residue detection.

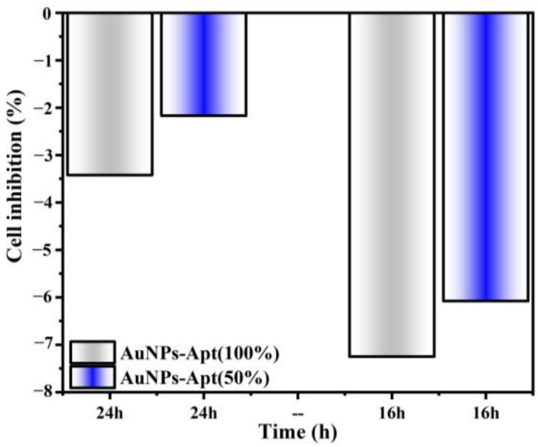

Figure S1. Cytotoxicity results of AuNPs-Apt (100 %) and AuNPs-Apt (50 %) after 16 and 24h incubation.

S2. EDS spectra

The EDS spectra and elemental contents of the sensing chip before and after functionalization are shown below

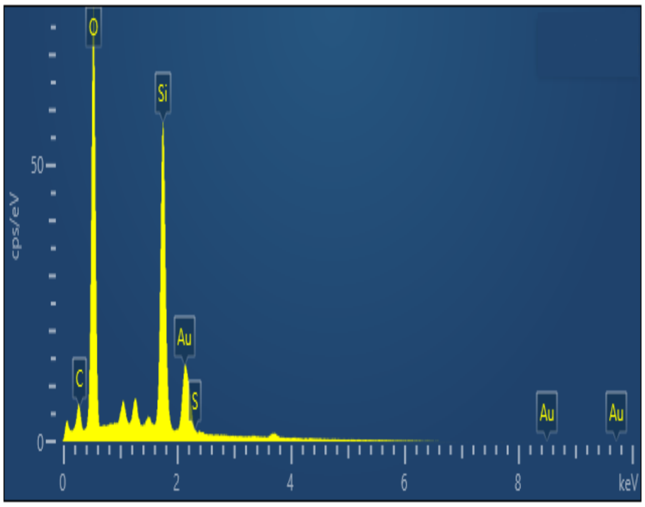

Figure S2. (a) EDS spectra after functionalization of sensing chips.

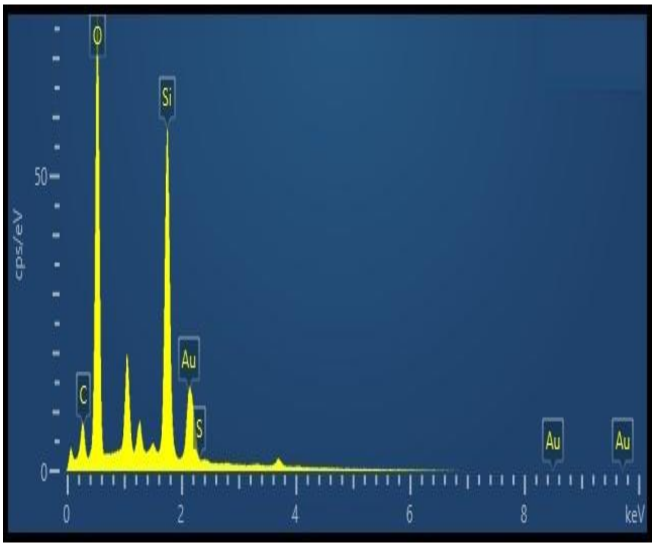

Figure S2. (b) EDS spectra before functionalization of sensing chip.

Table S1. The elemental contents of the sensing chip after functionalization.

Element	Apparent Concentration	Wt %
C	4.54	7.19
O	155.66	44.88
Si	61.49	23.90
S	0.70	0.34
Au	33.92	23.69
Total:		100.00

Table S2 The elemental contents of the sensing chip before functionalization.

Element	Apparent concentration	Wt %
C	5.50	8.98
O	142.01	43.91

Si	56.76	23.31
S	0.17	0.09
Au	32.19	23.72
Total：		100.00
